# Different Lung Parenchyma Quantification Using Dissimilar Segmentation Software: A Multi-Center Study for COVID-19 Patients

**DOI:** 10.3390/diagnostics12061501

**Published:** 2022-06-20

**Authors:** Camilla Risoli, Marco Nicolò, Davide Colombi, Marco Moia, Fausto Rapacioli, Pietro Anselmi, Emanuele Michieletti, Roberta Ambrosini, Marco Di Terlizzi, Luigi Grazioli, Cristian Colmo, Angelo Di Naro, Matteo Pio Natale, Alessandro Tombolesi, Altin Adraman, Domenico Tuttolomondo, Cosimo Costantino, Elisa Vetti, Chiara Martini

**Affiliations:** 1 Department of Radiological Function, “Guglielmo da Saliceto” Hospital, Via Taverna 49, 29121 Piacenza, Italy; camilla.risoli11@gmail.com (C.R.); d.colombi@ausl.pc.it (D.C.); m.moia2@ausl.pc.it (M.M.); fausto.rapacioli@gmail.com (F.R.); p.anselmi@ausl.pc.it (P.A.); e.michieletti@ausl.pc.it (E.M.); 2 Department of Diagnostic Imaging, Spedali Civili di Brescia, Piazzale Spedali Civili 1, 25123 Brescia, Italy; marco.nicolo.20@gmail.com (M.N.); robertambrosini@gmail.com (R.A.); mditer@gmail.com (M.D.T.); luigi.grazioli@asst-spedalicivili.it (L.G.); 3 Department of Radiology, Diagnostic Institute Antoniano Affidea, Via Cavazzana, 39/4, 35123 Padova, Italy; colmocristian@gmail.com; 4 Department of Oncology and Hematology, Papa Giovanni XXIII Hospital, Piazza OMS, 1, 24127 Bergamo, Italy; angelo.dinaro@unimib.it; 5 Department of Respiratory Disease, University of Foggia, Via Antonio Gramsci, 89, 71122 Foggia, Italy; matteo.natale@unifg.it; 6 Department of Radiology, University Hospital of Città della Salute e della Scienza di, 10127 Torino, Italy; alessandro.tombolesi@gmail.com; 7 Department of Radiology, Santa Chiara Hospital, Largo Medaglie d’oro, 9, 38122 Trento, Italy; a.altin1997@gmail.com; 8 Department of Cardiology, Parma University Hospital, Via Gramsci 14, 43125 Parma, Italy; d.tuttolomondo@hotmail.it; 9 Department of Medicine and Surgery, University of Parma, 43126 Parma, Italy; cosimo.costantino@unipr.it; 10 Department of Health Professions, University of Parma, Maggiore Hospital, Via Gramsci 14, 43125 Parma, Italy; evetti@ao.pr.it; 11 Department of Medicine and Surgery, Section of Radiology, University of Parma, Maggiore Hospital, Via Gramsci 14, 43125 Parma, Italy

**Keywords:** chest CT, lung segmentation, semi-automatic segmentation software, COVID-19 pneumonia, post-processing tools

## Abstract

Background: Chest Computed Tomography (CT) imaging has played a central role in the diagnosis of interstitial pneumonia in patients affected by severe acute respiratory syndrome coronavirus 2 (SARS-CoV-2) and can be used to obtain the extent of lung involvement in COVID-19 pneumonia patients either qualitatively, via visual inspection, or quantitatively, via AI-based software. This study aims to compare the qualitative/quantitative pathological lung extension data on COVID-19 patients. Secondly, the quantitative data obtained were compared to verify their concordance since they were derived from three different lung segmentation software. Methods: This double-center study includes a total of 120 COVID-19 patients (60 from each center) with positive reverse-transcription polymerase chain reaction (RT-PCR) who underwent a chest CT scan from November 2020 to February 2021. CT scans were analyzed retrospectively and independently in each center. Specifically, CT images were examined manually by two different and experienced radiologists for each center, providing the qualitative extent score of lung involvement, whereas the quantitative analysis was performed by one trained radiographer for each center using three different software: *3DSlicer*, *CT Lung Density Analysis*, and *CT Pulmo 3D*. Results: The agreement between radiologists for visual estimation of pneumonia at CT can be defined as good (ICC 0.79, 95% CI 0.73–0.84). The statistical tests show that 3DSlicer overestimates the measures assessed; however, ICC index returns a value of 0.92 (CI 0.90–0.94), indicating excellent reliability within the three software employed. ICC was also performed between each single software and the median of the visual score provided by the radiologists. This statistical analysis underlines that the best agreement is between 3D Slicer “LungCTAnalyzer” and the median of the visual score (0.75 with a CI 0.67–82 and with a median value of 22% of disease extension for the software and 25% for the visual values). Conclusions: This study provides for the first time a direct comparison between the actual gold standard, which is represented by the qualitative information described by radiologists, and novel quantitative AI-based techniques, here represented by three different commonly used lung segmentation software, underlying the importance of these specific values that in the future could be implemented as consistent prognostic and clinical course parameters.

## 1. Introduction

Since December 2019, a significant number of novel pneumonias with unknown etiology has been registered in Wuhan, Hubei province, China [1]. In January 2020, a new type of coronavirus capable of infecting humans was identified and referred to as “Severe Acute Respiratory Syndrome Coronavirus 2” (SARS-CoV-2). Previous research demonstrated that this virus belongs to the species of SAR-related coronaviruses [2]. SARS-CoV-2 is responsible for a different series of clinical outcomes, from mild flu-like symptoms to severe pneumonia, which can be complicated by serious dyspnoea [3]. On 11 March 11 2020, the World Health Organization (WHO) declared COVID-19 (SARS-CoV-2 related disease) as a pandemic [4], the first pandemic ever to be caused by a coronavirus that importantly prompted extensive clinical research and subsequent follow-up studies [5].

Notably, radiology has played a central role in the diagnosis of interstitial pneumonia caused by COVID-19, especially using chest Computed Tomography (CT) imaging [6]. In COVID-19 pneumonia disease, extension percentages of regularly well-aerated and pathologic lungs have shown a prognostic value [7,8,9]. These percentages can be visually evaluated on the chest CT images or also calculated by Artificial Intelligence (AI)-powered software that elaborate and compute auto/semiautomatic lung-CT image segmentation, providing a numerical analysis [10], which could enhance the qualitative visual evaluation during the medical image report step performed by radiologists. The segmentation is grounded on different Hounsfield Unit (HU) thresholds: these identify different densities and therefore different tissues [11,12]. According to the literature [7,9,11], the range between [−700; −750 HU] has been identified as the optimal starting threshold to distinguish the COVID-19 lesions and characterize the disease. During this pandemic, a lot of software sharing the same goal but with different characteristics has been developed to support the radiological workflow.

This study has two main aims. Primarily, the percentage values of the pathological lung extension visually determined by radiologists were associated and compared to the quantitative percentage results returned by the three different software employed for lung segmentation (3DSlicer, CT Lung Density Analysis, and Syngo CT Pulmo 3D) [13,14,15]. Consequently, the numerical results for each software were analyzed to verify their agreement.

## 2. Materials and Methods

### 2.1. Study Population

This retrospective study was approved by the Local Ethics Committee (protocol approval number 553/2021/OSS/AUSLPC-LUNG-COVID-19).

In total, 120 patients were included in this study: 60 patients from Piacenza Hospital (“Guglielmo da Saliceto” Hospital, called in this study “Center 1”) and 60 patients from Brescia Hospital (“Spedali Civili” Hospital, renamed in this paper as “Center 2”), previously anonymized to ensure their privacy. These 120 patients were randomly selected from an original pool of patients with a defined diagnosis of COVID-19 validated by reverse-transcription polymerase chain reaction (RT-PCR) nasal-pharyngeal swabs for SARS-CoV-2 detection and who underwent a chest CT for COVID-19 pneumonia (as initial diagnosis or follow-up) ordered by the emergency department or hospital wards between November 2020 and February 2021 (the so-called “the second wave” by the Italian statement). CT scans conducted without the use of contrast enhancement were considered. Particularly, patients concerned in this study had both at least one positive RT-PCR test within 48 h of being admitted to the hospital and a CT scan conducted within 12 h of the clinical examination and laboratory tests.

### 2.2. CT Protocol

For both patients studied in Center 1, unenhanced chest CT was performed in the supine position during an inspiratory breath-hold, moving from the apex to the lung bases. In Center 1, CT acquisition was performed using a 16-slice scanner (Emotion 16; Siemens, Forchheim, Germany) with the following parameters: tube voltage set at 130 kV; tube current: fixed mAs depending on the patient body weight [16,17]; pitch: 0.8 or more, adjusted on the seriousness of patient dyspnoea; collimation: 1.2 mm; and thickness and increment: 2 mm. After each examination, the room was decontaminated with a solution of 62–71% of ethanol or 0.1% of sodium hypochlorite [11]. The scanner cleaning procedure required around 15–20 min for each patient. Image data sets were reconstructed using both sharp kernels (B70f) with standard lung window settings (window width, 1500 HU; window center, −50 HU) and medium-soft kernels (B40f) with soft-tissue window settings (window width, 300 HU; window center, 40 HU).

Meanwhile, in Center 2, CT acquisition was performed using two different types of CT equipment. The examination with the 80-slice CT scanner (Aquilion PrimeSP, Canon Medical System Europe B.V, Zoetermeer, The Netherlands) was performed with: 120 kVp; mAs based on the Automatic Exposure Control system (AEC) with Standard Deviation (SD) of 12.5 [18]; pitch 0.8 or higher to speed the acquisition up in case of severe dyspnoea; 0.5 mm collimation; 2 mm slice thickness and increment; and sharp kernel (FC56) and medium-soft kernels (FC08 and FC18). 

The scans with a 64-row CT scanner (Somatom Go.Top, Siemens, Forchheim, Germany) were performed with the following parameters: range of kV 110-120-Sn110 and -Sn140 (Sn = tin filter); mAs based on the AEC with the following Quality Reference mAs: 10-60-100-110 [18]; pitch set on 0.8 or higher to reduce the scan time; collimation of 0.625 mm; the same slice thickness and increment cited earlier (2 mm); a medium-soft kernel (Br40) and a sharp kernel (Br60) for a soft-tissue window (window width, 300 HU; window center, 40 HU) and lung window (window width, 1500 HU; window center, −500 HU), respectively.

The technical parameters are recorded in Table 1.

### 2.3. Image Analysis

For this study, visual chest CT interpretation was independently performed by four radiologists (D.C. and F.R., with 5 and 2 years of experience, respectively, for Center 1; R.A. and M.D, with more than 10 years of experience, respectively, for Center 2). They knew that the patients were affected by COVID-19 pneumonia, but they were blinded to the previous medical report. Each observer identified the extension of the COVID-19 disease in terms of visual percentage, consisting of ground-glass opacity (GGO), crazy-paving pattern (CP), and consolidation (CONS) as defined by the Fleischner Society Glossary of terms for Thoracic Imaging [19], from both lungs.

Then, the reader categorized CT findings as proposed by Sverzellati et al. [20]: the total extent of COVID-19 pneumonia, defined as the GGO and CP sum (GGO + CP) and CONS were expressed as a percentage of the total lung volume and averaged to produce a global percentage of abnormalities extent [20]. Consensus formulation for the visual scores and categorical CT assessment was defined in previous studies [9].

The software-based evaluation was performed on a dedicated workstation using three different software:

- Center 1: the extension “Chest Imaging Platform” of the open-source 3DSlicer software (version 4.11.2). In this extension, two different kinds of analysis programs were used: “Parenchyma Analysis” and “LungCTAnalyzer” [13,21].
- Center 2: CT Lung Density Analysis software by Canon (version 7.11.5) [14] and Syngo.CT Pulmo 3D by Siemens (version VB30) [15].

**Figure 1:**
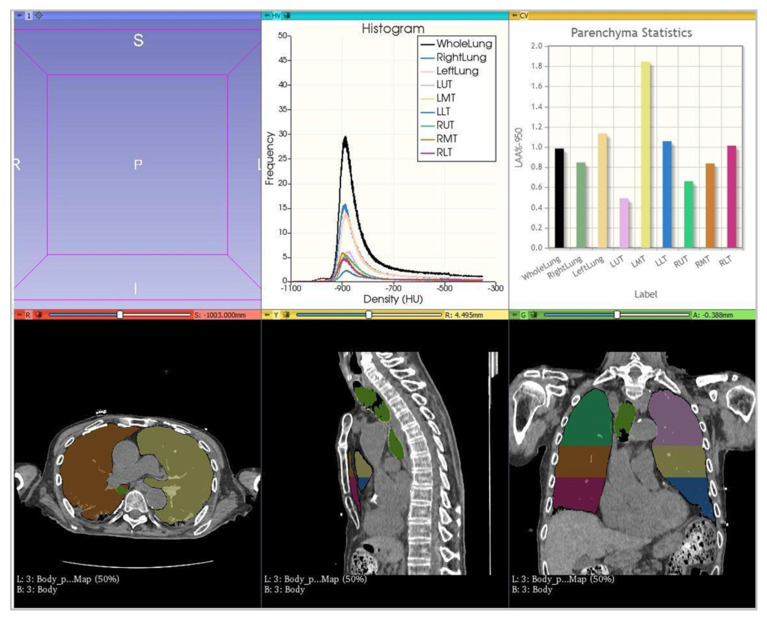
Lung segmentation provided by 3DSlicer. In the first stripe, two graphics concerning the extensions of different densities are shown. In the second stripe, there are three representations of the lung segmentation as provided by the 3DSlicer software, in axial, sagittal, and coronal multiplanar reconstructions.

**Figure 2:**
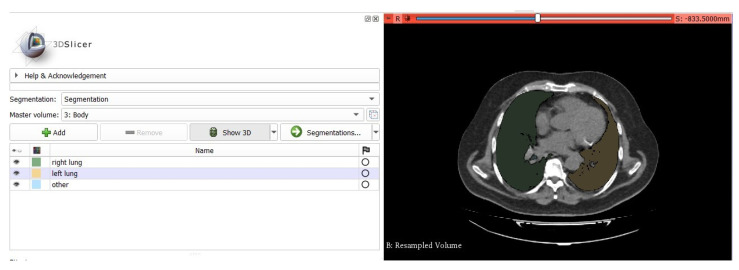
The 3DSlicer manual tool (“*Segment Editor*”) accomplished the unsatisfactory segmentation. In this section, the user can perform a manual segmentation using the colors listed on the left board.

For the 3DSlicer, “Parenchyma Analysis”, a fully automatic lung segmentation and analysis of lung parenchyma histogram, was obtained using a B40f kernel (Figure 1). A radiographer (C.R.) with 5 years of experience and precisely well-trained for the software employment [22] accomplished the segmentation of each patient and, if unsatisfactory, amended the lung contours with the manual tool “Segment Editor” (Figure 2). The time employed for the manual correction was recorded.

The percentage of the total lung volume with attenuation higher (High Attenuation Area, HAA) than −700 HU (%HAA −700), −600 HU (%HAA −600), −500 HU (%HAA −500), and −250 HU (%HAA −250) were recorded. Furthermore, the percentage of Low Attenuation Area (LAA) less than −950 HU (%LAA −950), −925 HU (%LAA −925), −910 HU (%LAA −910), −856 HU (%LAA −856) was additionally provided. These thresholds were fixed by default and non-editable. Hence, in this case, the area under the curve (AUC) within the range of −700 HU and 0 HU was used to quantify the lung parenchyma involved and affected by the disease.

Other measurements were obtained using the 3DSlicer “LungCTAnalyzer” [13,21]: after importing the CT datasets (medium-soft kernel B31s), lung masks were automatically segmented by the “LungCTSegmenter” extension [23] using 13 manually marked points. Three points were placed in axial and coronal views inside the right and the left lung and one point in the trachea. All lung masks were verified visually, and slight corrections were manually done using selected tools from the “Segment Editor”. The time employed for the manual correction was recorded. LungCTAnalyzer was then used to quantify “emphysematous”, “inflated”, “infiltrated”, and “collapsed” lungs in millilitres and percentage. Additionally, intrapulmonary vessels were segmented and subtracted from the aforementioned lung segments. There was no compensation for intrapulmonary bronchi.

**Figure 3:**
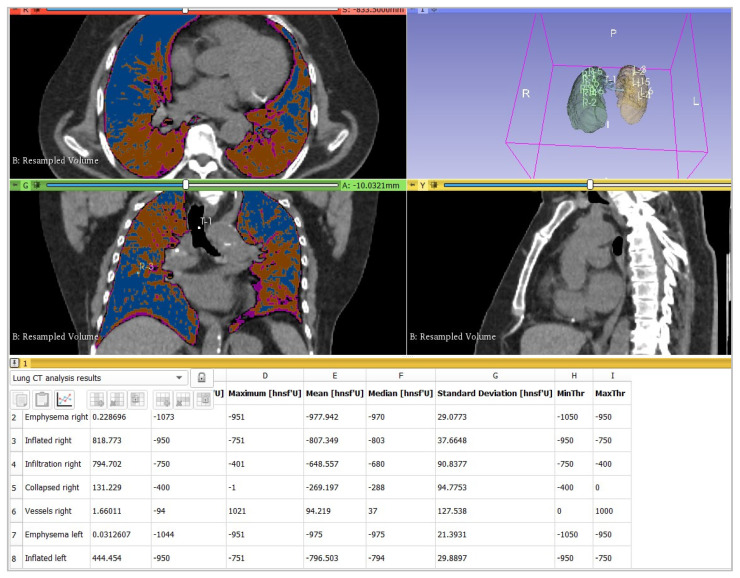
LungCTAnalyzer and the thresholds used. Here is an example of the analysis pursued. It was quantified that the “emphysematous”, “inflated”, “infiltrated”, and “collapsed” lung parenchyma were affected, and also their respective percentages were calculated.

Each category mentioned below (emphysematous, inflated, infiltrated, collapsed) has their own range of HU: emphysematous [−1050; −950 HU], inflated [−950; −750 HU], infiltrated [−750; −400 HU], and collapsed [−400; 0 HU] (Figure 3). Theoretically, these thresholds are variable, and the user may manually modify them. Nevertheless, according to the literature [7,9,11], the thresholds set by default were already optimized and so were easily comparable with the thresholds used by the other software employed in this study. Therefore, the AUC within the range of [−750 HU; 0 HU] was collected to quantify the lung parenchyma involved in the COVID-19 disease. After the analysis, the software returns a table with some percentages: particularly, the column “Involved” represents the total amount of the extension of the COVID-19 disease.

**Figure 4:**
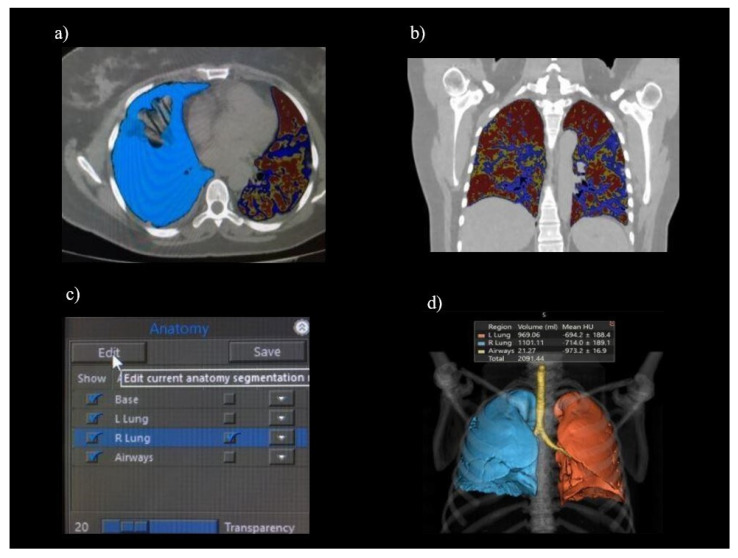
CT Lung Density Analysis. This image shows: (**a**) parenchyma contouring; (**b**) lesion display; (**c**) manual editing; (**d**) final result with a volumetric rendering.

**Figure 5:**
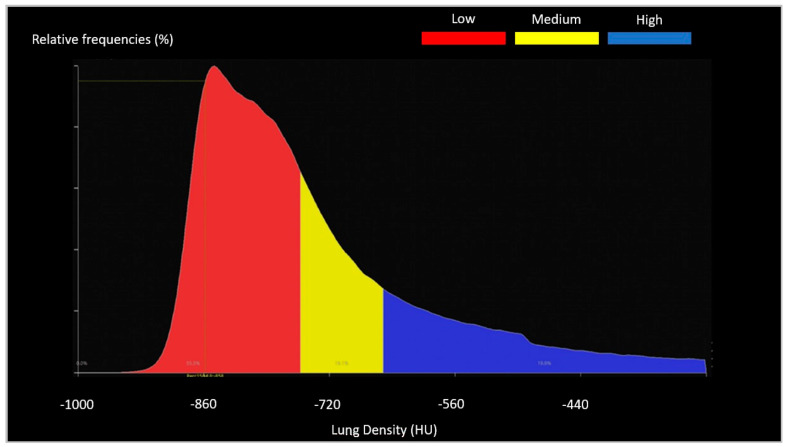
CT Lung Density Analysis: histogram of attenuation areas. The red area represents the low attenuation area [−990; −750 HU]; the yellow one represents the medium attenuation area [−750; 660 HU]; and the blue area shows the high attenuation area [−660; 0 HU].

For both software used in the Center 2, a radiographer (M.N.) with 5 years of experience and well-trained regarding the subject matter performed the lung segmentation of each patient; manual editing was also permitted at the end of the automatic segmentation provided by the software to better contour the lung parenchyma involved in case of unsatisfying segmentation (Figure 4). Manual settings of the HU threshold were available. CT Lung Density Analysis used a medium-soft kernel (FC08) to start the process and the HU threshold was set to [−990; −750 HU] (low attenuation area), [−750; −660 HU] (medium attenuation area), and [−660; 0 HU] (high attenuation area). The attenuation areas are shown in Figure 5.

**Figure 6:**
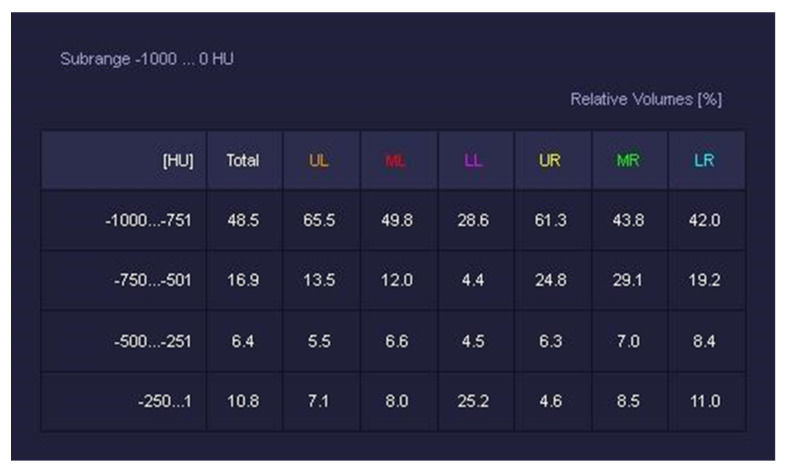
CT Pulmo 3D: thresholds and volumes. This figure shows the HU thresholds and relative volumes per lobe.

**Figure 7:**
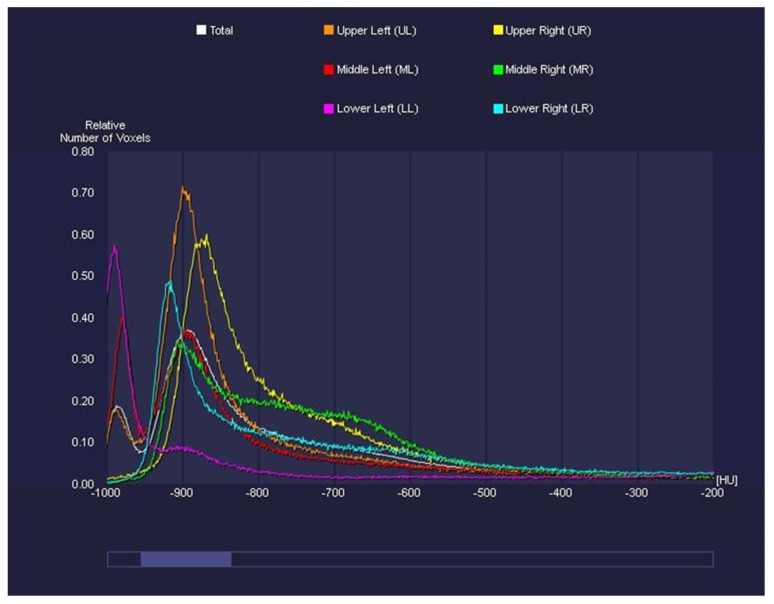
CT Pulmo 3D: graphic of HU thresholds. This graphic represents the HU thresholds and their relative frequencies.

**Figure 8:**
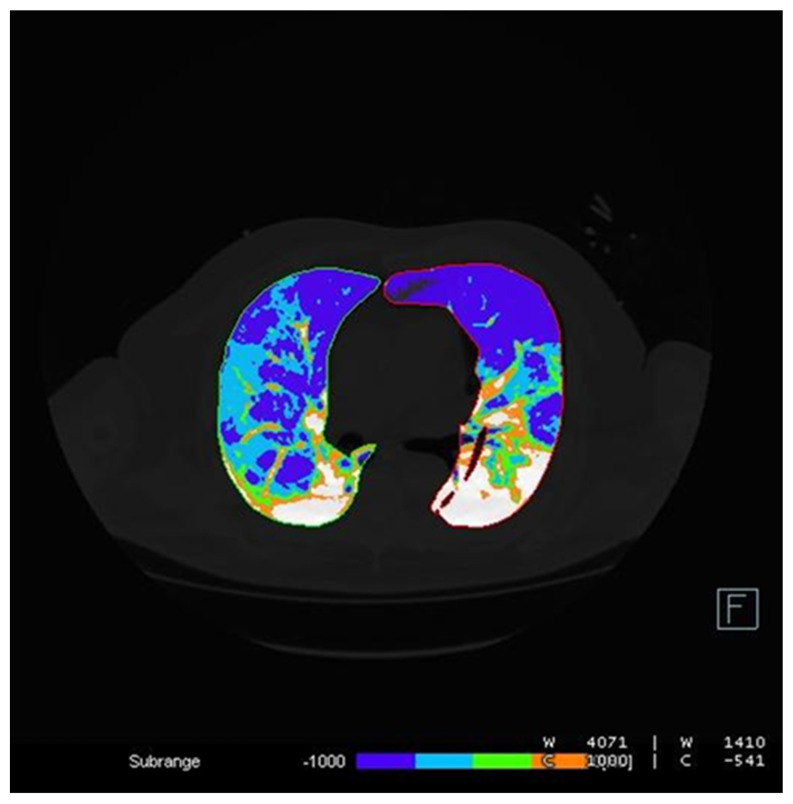
CT Pulmo 3D: lung contouring. This figure shows the lung parenchyma lesions counted; the different colors employed suggest different HU sub-ranges.

CT Pulmo 3D involved a sharp kernel (Br60) for the lung segmentation and four ranges of HU were set: first range [−1000 HU; −750 HU]; second range [−750 HU; −550 HU]; third range [−550 HU; −249 HU]; and fourth range [−249 HU; 0 HU] (Figure 6). For either software, a histogram of lung parenchyma was calculated and the area equal to or less than −750 HU was considered affected and involved based on previous studies (Figure 7 and Figure 8) [22,24].

### 2.4. Statistical Analysis

The statistical calculation has been assessed with the software MedCalc (https://www.medcalc.org/ accessed on 23 February 2022). Categorical and continuous variables were expressed as counts and percentages or median with a corresponding 95% confidence interval (95% CI).

The ICC (Intraclass Correlation Coefficient) statistical index was used to establish agreement within the group of the four radiologists on the visual score for the extension of the disease on the CT images and within the three software assessments about the extension of disease. Following ICC interpretation [25], values less than 0.50 are indicative of poor reliability, values between 0.50 and 0.75 indicate moderate reliability, values between 0.75 and 0.90 indicate good reliability, and values greater than 0.90 indicate excellent reliability.

Moreover, a Friedman test was performed; it is a non-parametric test used to examine the differences between groups when the dependent variable being measured is ordinal [26]. It was used to determine whether there is a statistically significant difference between the means/medians of three or more groups in which the same subjects show up in each group.

## 3. Results

### 3.1. Patient Characteristics

The mean age of the 120 patients (male 63) enlisted in this study was 70 ± 14 years (range 42–92). The interquartile range (IQR, 25° and 75°) was, respectively, 60 and 81.

### 3.2. Relationships and Inter-Reader Agreement of Visual and Software-Based CT Assessment

**Figure 9:**
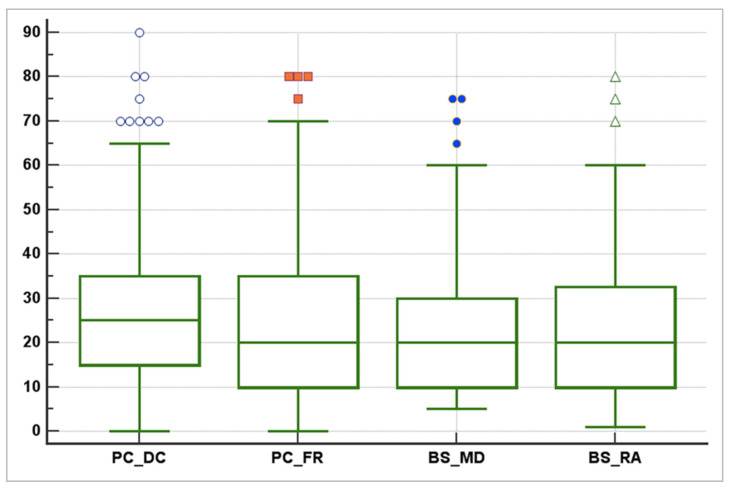
The descriptive statistics from the radiologists’ measurements. The agreement between radiologists for visual estimation of pneumonia from CT was good, as shown in this box, plot scheme (ICC 0.79, 95% CI 0.73–0.84). Only one radiologist esteemed a higher visual score (considered as the median). ICC = Intraclass Correlation Coefficient, CI = Confidence Interval.

The agreement between radiologists for visual estimation of pneumonia at CT was good (ICC 0.79, 95% CI 0.73–0.84); only one radiologist (D.C.) esteemed a higher visual score (considered as the median), as shown in the box plot scheme (Figure 9). Table 2 summarises the descriptive statistic managed within the radiologists’ measurements.

**Figure 10:**
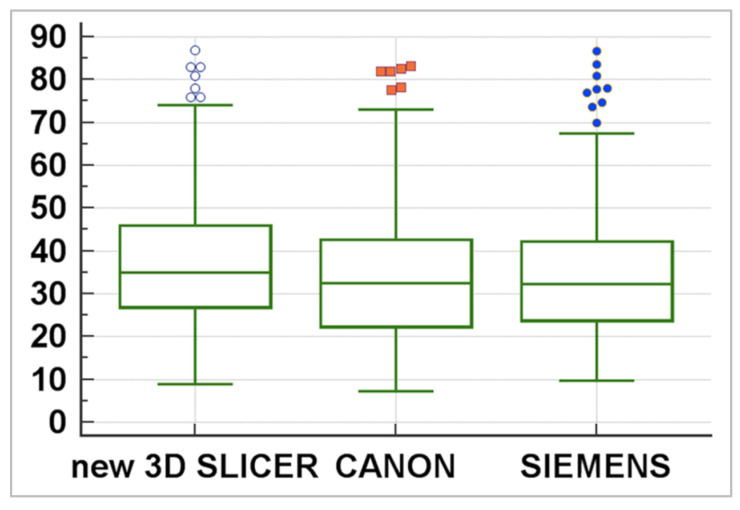
Statistical description of the three software. This box plot scheme shows that 3DSlicer delivers a higher value in the median of the measures assessed.

The statistical tests performed between the three software showed that the 3DSlicer delivers a higher value in the median of the measures assessed (Figure 10), whereas the ICC index returned a value of 0.92 (CI 0.90–0.94), indicating excellent reliability within the three software employed. Table 3 summarises the statistical characteristics of the values obtained with these three software.

ICC was also performed between each single software and the median of the visual score provided by the radiologists. This statistical calculation underlined that the best agreement was between the 3DSlicer “LungCTAnalyzer” and the median of the visual score (0.75 with a CI 0.67–0.82 and with a median value of 22% of disease extension for the software and 25% the visual values). In order, the comparison shows in second place the Canon software, in third place the Siemens software and, finally, the 3DSlicer version “Parenchyma Analysis”. The data of these evaluations and the ICC indexes are reported in Table 4. It must be noticed that all three-software overestimate the extension of COVID-19 pneumonia, except 3Dslicer “Parenchyma Analysis”, which is characterized by a lower median score.

**Figure 11:**
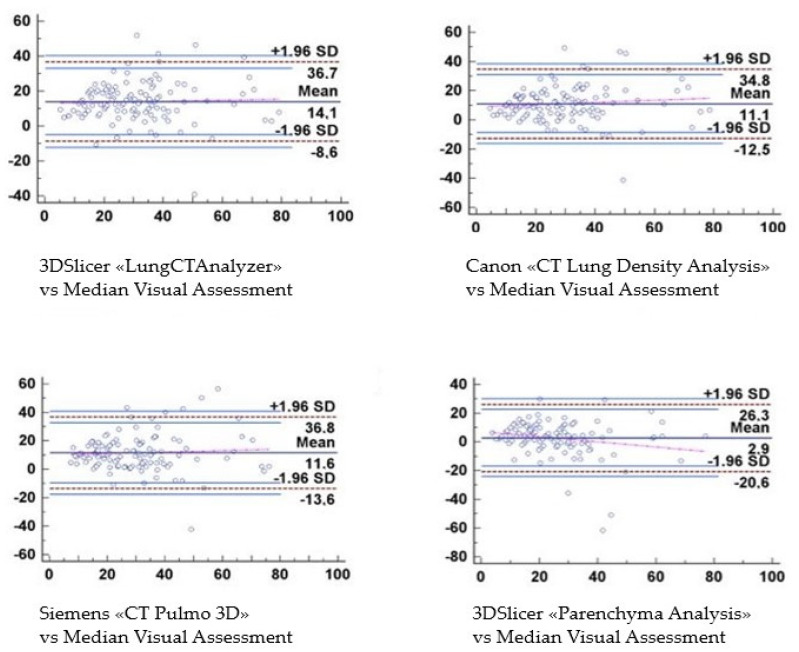
Bland–Altman graphics demonstrate the trend of the values assessed. For the software “LungCTAnalyzer”, the results lie in a range between 36.7 and −8.6 with an SD of ±1.96. For the Canon software, the results filled the space between 34.8 and −12.5 with an SD of ±1.96; for the Siemens software, instead, the values were positioned between 36.8 and −13.6 with the same SD of ±1.96. Finally, the “Parenchyma Analysis” outcomes were placed between 26.3 and −20.6 with an SD of ±1.96. SD = Standard Deviation.

A final Bland–Altman graphic was performed to better demonstrate the evolution of the results obtained with the three software (Figure 11). For the software “LungCTAnalyzer”, the results lie in a range between 36.7 and −8.6 with an SD of ±1.96. For the Canon software, the results filled the space between 34.8 and −12.5 with an SD of ±1.96; for the Siemens software, instead, the values were positioned between 36.8 and −13.6 with the same SD of ±1.96. Finally, the “Parenchyma Analysis” outcomes were placed between 26.3 and −20.6 with an SD of ±1.96.

## 4. Discussion

This study has shown agreement among radiologists upon visual reporting, except for one radiologist who overestimated the extent of lung parenchyma involved.

Furthermore, the extent of concordance of the three analyzed software was excellent, despite all three software having presented what seems to be an overestimation, in particular, the 3D Slicer “LungCTAnalyzer’’. However, the 3DSlicer “LungCTAnalyzer” also showed the best agreement between its assessments and the visual score produced by the radiologists. In addition, when ICC was calculated between each software and the median visual score, it was noticed that ICC seems to become higher when the median software assessment obtains a higher value.

Numerous studies have outlined the use of unenhanced chest CT to better define and analyze lung parenchyma affected by Sars-CoV-2. A typical COVID-19 pattern is often presented with peripheral and bilateral ground-glass opacities (GGO) and bilateral patchy shadowing [25]. The evaluation of the parenchyma involved in pneumonia may be obtained following different guidelines. For example, Ruch Y. et al. [27] suggested the analysis of lung parenchyma involved classified as percentage in six different groups: normal CT (no lesion), minimal (0–10%), moderate (11–25%), important (26–50%), severe (51–75%), and critical (>75%). In this paper, we have followed Simpson et al. [28] that suggested four categories (typical appearance, indeterminate appearance, atypical appearance, negative for pneumonia), each with standardized language, to make a structured report when it comes to diagnosing COVID-19 disease and helping to manage clinical decisions.

In our study, all three software have displayed overestimation during the evaluation of the concordance between visual score and their quantitative results. Particularly, the Bland–Altmann graphics underlined that the 3DSlicer “LungCTAnalyzer’’ tends to overestimate the values from 20% of the disease extension forward. Canon’s and Siemens’ software showed a comparable trend too.

The explanation could be found in the vessels and partially bronchi considered by the three software. Visual proof had been assessed using the tool “Segment Editor” of the 3DSlicer; in fact, this tool allows the user to take off each single part of the segmentation. In this way, the user can decide to see only one segmented part of the lung at a time. By doing this, the text had shown that the software segmented not only the vessels and bronchi themself but also a large area around them in an inexplicable way. This may be the reason for the overestimation, even if we do not have objective evidence. Further studies should investigate these features in the future.

Nevertheless, the 3DSlicer “Parenchyma Analysis” has shown an underestimation but the reason is quickly explained; in fact, it uses a fixed threshold put on −700 HU to describe the extension of the COVID-19 disease. This threshold is in contrast with some of the literature evidence, which suggests setting the HU threshold for detecting GGO opacities at −750 HU [29].

Alongside this, the implementation of automatic or semi-automatic segmentation software might make the reporting process smoother. The most similar study found in the literature is the one written by Grassi et al. [30], which examined three different software by three different vendors. They compared the ability of this software to quantify pneumonia lesions in COVID-19 infections to stratify the patients based on output coming from the software and radiologists. A peculiarity to highlight is that every software used a different HU threshold to quantify the lung parenchyma involved, whereas in this current study, the threshold was set to the same level. Moreover, Ippolito et al. [24] evaluated the ability of software (COPD, Intellispace Portal, Philips Healthcare) to properly detect and quantify lung volume quantification in Sars-Cov-2-related pneumonia. The HU threshold set for affected lung parenchyma was −700 HU and the CT images were also analyzed by two radiologists using the score proposed by Huang et al. [31], which is based on the extent of GGO involvement in the lobes. The maximum score possible was 5 for each lobe involved (1 less than 5%; 2, 5–25%; 3, 26–49%; 4, 50–75%; and 5, more than 75%). Lastly, CT findings of crazy-paving or consolidation in each lobe would have increased the score by 1. In the end, Colombi et al. [11] assessed the association between death and both qualitative and quantitative CT parameters obtained either by software or visually. The visual score was evaluated according to the Fleischner Society Glossary of terms for Thoracic Imaging [19] and the software analysis was obtained by 3D Slicer software with previous releases that had no adjustable HU thresholds.

Shen C. et al. [32] compared an in-house software with a visual score obtained by two radiologists. The score could be summed up as 0–3 for each lobe: 0, no lesion present, 1, 1 < ⅓ involvement, 2, >⅔ and <⅔ involvement, and 3, >⅔ involvement. Their scope was to stratify the severity of COVID-19 based on unenhanced chest CT images. The results showed a strong or moderate correlation between the lesion percentage obtained by radiologists and the computer software, a negative correlation between the proportion of GGO and mean lesion density, and a moderate positive correlation between the proportion of consolidation and mean lesion density. This indicates that computer tools might be reliable in assessing COVID-19 lesions.

Therefore, as far as our knowledge extends, these are the studies that investigated the ability of automatic or semi-automatic AI-based software to properly quantify the amount of lung parenchyma affected by Sars-CoV-2 compared to visual scores by radiologists.

Future implications of using computer tools might be implemented with fully automatic AI-based quantification software, such as Li L 2020 et al. [33] and Jungmann F. et al. [34] suggested in their study. This last one calculated the sensitivity, specificity, positive predictive value (PPV), the negative predictive value (NPV), and the area under the curve (AUC) for four AI solutions from different vendors compared to the visual score obtained by two radiologists to differentiate Sars-CoV-2 related pneumonia from other lung conditions. Their results highlighted low specificity (31–80%), whereas the AUC was >0.70 for three companies, and 0.54 for the other company in the analysis of all CT studies with proven COVID-19 pneumonia. PPV in this study ranged between 0.19 and 0.25. Therefore, the authors suggested AI has the potential to support radiologists in their daily practice, but further randomized trials are needed to better evaluate AI’s benefit since many patients might be analyzed with false positives. Moreover, as shown by Galli M.G. et al. [35], quantifying the lung parenchyma affected by Sars-Cov-2 and knowing the time from symptom onset are the main determinants of hospital readmission in COVID-19 patients, resulting in a useful and helpful implementation of AI-based software for the future.

The limitations of this paper lie in the retrospective nature of the study and, more importantly, in having just one radiographer per center analyzing patients with the segmentation software. In fact, sometimes the software is not able to perform a precise segmentation of the CT images, particularly if the lungs contain spill or many consolidations. In these cases, the software may fail the correct segmentation and it represents a disadvantage in the use of this device, of course. In such circumstances, a manual segmentation occurs but it is a time-consuming process, which can be defined as “operator-based” and, therefore, highly trained and specialized radiographers are needed.

Lastly, since the employed software presents adjustable thresholds for the description of specific densities, for example, the HU threshold to identify the well-aerated lung parenchyma is not the same across the software, and so it is important to set the same HU threshold as a reference. In fact, the same HU threshold for detecting affected lung parenchyma was set in this study.

## 5. Conclusions

This study has proved that there is an agreement between the visual score provided by the radiologists and the quantitative score assessed by the semi-automatic software involved in this study. Moreover, the three considered software have shown excellent concordance, with a slight overestimation of the extension of the disease, in comparison with the qualitative assessment of the visual score. This means that these software could be used in daily routines to help the radiologist work and make the reporting workflow smoother in a radiology unit. Moreover, they are eligible to support the radiologists during their daily reporting activity.

Overall, these software are not able to replace them but they might support their work activities. In particular, radiographers should be the well-educated healthcare professionals able to support the radiologist’s work using this software and facilitate the reporting process.

Take home messages:

- Semiautomatic segmentation software should be employed in daily work activities to make the reporting workflow smoother.
- Different software have excellent concordance, so their employment is reliable and practicable in daily routines.

## Figures and Tables

**Table 1:** Technical parameters of the CT protocols. The Table 1 summarizes and compares the technical parameters set on the three CT scans employed in this study to obtain chest images.

	Rows	kV	mAs	Pitch	Collimation	Thickness	Increment
**Emotion 16**	16	130	fixed, adjusted on the patient body weight	>0.8	1.2 mm	2	2
**Aquilion PrimeSP**	80	120	AEC with SD 12.5	>0.8	0.5 mm	2	2
**Somatom Go.Top**	64	100-110-Sn110-Sn140	AEC with Q.ref.mAs 10-60-100-110	>0.8	0.625 mm	2	2

**Table 2:** Summary of descriptive statistical calculation obtained from the four radiologists. This table shows good reliability among the four radiologists, as underlined by the good ICC score (0.79). ICC = Intraclass Correlation Coefficient.

	25th Percentile	Median	75th Percentile
**1° radiologist (D.C.)**	15	25	35
**2° radiologist (F.R.)**	10	20	35
**3° radiologist (M.D.)**	10	20	30
**4° radiologist (R.A.)**	10	20	32.5
**ICC = 0.79**			

**Table 3:** Summary of descriptive statistical calculation within the three software. The ICC index returned a value of the reliability of 0.92 (CI 0.90–0.94), indicating an excellent one within the three software employed. ICC = Intraclass Correlation Coefficient, CI = Confidence Interval.

	25th Percentile	Median	75th Percentile
**3DSlicer**	27	35	46
**Canon**	22	32	43
**Siemens**	24	32	42
**ICC = 0.92**			

**Table 4:** Summary of the evaluations within the software and the visual assessments. This statistical calculation underlines that the best agreement was between 3DSlicer “LungCTAnalyzer” and the median of the visual score (ICC 0.75 with a CI 0.67–0.82 and with a median value of 22% of disease extension for the software and 25% for the visual values). The Canon software occupies second place, the Siemens software third place, and the 3DSlicer version “Parenchyma Analysis” last place. ICC = Intraclass Correlation Coefficient, CI = Confidence Interval.

Software Name	Vendor	Median Software Assessment	Median Visual Assessment	ICC
**LungCTAnalyzer**	**3DSlicer**	35	23	0.75
**CT Lung Density Analysis**	**Canon**	32	23	0.74
**CT Pulmo 3D**	**Siemens**	32	23	0.70
**Parenchyma Analysis**	**3DSlicer**	25	23	0.69
